# Multipotent stem cells of the heart—do they have therapeutic promise?

**DOI:** 10.3389/fphys.2015.00123

**Published:** 2015-05-08

**Authors:** Camila F. Leite, Thalles R. Almeida, Carolina S. Lopes, Valdo J. Dias da Silva

**Affiliations:** Department of Biochemistry, Pharmacology, Physiology and Molecular Biology, Institute for Biological and Natural Sciences, Triângulo Mineiro Federal UniversityUberaba, Brazil

**Keywords:** cardiac stem cells, cardiac remodeling, cell therapy, regenerative process, cardiac homeostasis

## Abstract

The last decade has brought a comprehensive change in our view of cardiac remodeling processes under both physiological and pathological conditions, and cardiac stem cells have become important new players in the general mainframe of cardiac homeostasis. Different types of cardiac stem cells show different capacities for differentiation into the three major cardiac lineages: myocytes, endothelial cells and smooth muscle cells. Physiologically, cardiac stem cells contribute to cardiac homeostasis through continual cellular turnover. Pathologically, these cells exhibit a high level of proliferative activity in an apparent attempt to repair acute cardiac injury, indicating that these cells possess (albeit limited) regenerative potential. In addition to cardiac stem cells, mesenchymal stem cells represent another multipotent cell population in the heart; these cells are located in regions near pericytes and exhibit regenerative, angiogenic, antiapoptotic, and immunosuppressive properties. The discovery of these resident cardiac stem cells was followed by a number of experimental studies in animal models of cardiomyopathies, in which cardiac stem cells were tested as a therapeutic option to overcome the limited transdifferentiating potential of hematopoietic or mesenchymal stem cells derived from bone marrow. The promising results of these studies prompted clinical studies of the role of these cells, which have demonstrated the safety and practicability of cellular therapies for the treatment of heart disease. However, questions remain regarding this new therapeutic approach. Thus, the aim of the present review was to discuss the multitude of different cardiac stem cells that have been identified, their possible functional roles in the cardiac regenerative process, and their potential therapeutic uses in treating cardiac diseases.

The increase in the average human lifespan has been accompanied by an increased incidence of chronic diseases (Torella et al., 2007). These chronic non-communicable diseases are the result of a number of risk factors and are characterized by a prolonged natural course and the potential for developmental disabilities. These diseases are not only prevalent but also aggressive, and they dominate death statistics. According to the World Health Organization (WHO), cardiovascular diseases (including heart attack and stroke) represent the largest number of deaths from non-communicable diseases, 17.5 million people annually, followed by cancer (8.2 million) and respiratory diseases (4 million)^1^.

Heart failure is a particularly lethal, disabling, and expensive-to-treat disorder that has reached epidemic levels in industrialized nations (Bolli et al., 2011). Even with significant advances in the treatment of coronary artery disease and the management of acute myocardial infarction in humans, this pathological event remains the leading cause of mortality in developed countries (Gnecchi et al., 2008; Wang et al., 2014) and is highly associated with morbidity because a large population of infarct survivors will develop cardiac failure (Hellermann et al., 2002). In addition to a significant level of morbidity, and direct cost of treatments by physicians and other professionals, hospital services, medications, home health care, and other medical durables, the health-related expenditures associated with cardiovascular diseases include indirect costs, such as lost productivity resulting from morbidity and premature mortality (Go et al., 2013). If all forms of major cardiovascular disease were eliminated, human life expectancy might increase by approximately 7 years (Go et al., 2014).

Given the striking impact of these diseases on mortality rates, most prognostic models of heart failure focus on mortality, which is easily determined and highly relevant; however, other clinical outcomes also rank high in importance to individual patients (Allen et al., 2012). Because a large fraction of patients with advanced heart failure report poor quality of life and significant emotional distress due to debilitating symptoms such as breathlessness, fatigue, and sleeping difficulties (Hallas et al., 2011), quality of life is a significant outcome to be considered. When advanced heart failure patients discuss their goals, quality of life is an important issue because how well they will live is as important as how long they will live (Allen et al., 2012).

Depending on the specific condition(s), therapeutic options for patients living with heart diseases include lifestyle changes, drugs to prevent disease advancement, angioplasty, bypass surgery, pacemakers, left ventricular assist devices and, finally, heart transplantation (Smith et al., 2008a). This final treatment is unavailable to many patients because of a lack of donor organs or the presence of comorbidities that make the procedure inviable.

Given the deleterious consequences of heart disease, new therapeutic approaches are needed because the progression of heart disease involves loss of the myocardium, scar formation and remodeling of remaining cardiac tissue (Smith et al., 2008a). Invasive treatments and optimized medical therapies are increasingly successful in addressing the acute manifestations of coronary artery diseases. However, although these treatment strategies often extend the lives of patients, they may not return to healthy lives (Torella et al., 2007). As the incidence of heart failure increases exponentially, novel therapies using cellular treatments or regenerative strategies are being sought (Chong et al., 2011). In particular, patients have high expectations for cellular therapy (Lovell and Mathur, 2011).

## Cardiac physiological homeostasis—the heart as a dynamic organ

Fortunately, the last decade has witnessed the dawn of a new era in myocardial biology (Ellison et al., 2012), with the initiation of many promising studies of treatment strategies based on cellular therapies for heart patients. Until recently, the heart was considered a static and post-mitotic organ lacking regenerative capacity (Beltrami et al., 2003; Chan et al., 2009; Lovell and Mathur, 2011; Ellison et al., 2012), i.e., the number of cardiomyocytes in an individual was established at birth (Barile et al., 2007; Kajstura et al., 2010b), and cardiomyocyte hypertrophy was considered the only cellular adaptive response of the heart (Lovell and Mathur, 2011). In this context, it was assumed that the age of a cardiomyocyte would correspond to the age of the organism (Torella et al., 2007; Kajstura et al., 2010a) and that a cardiomyocyte, lacking the ability to divide, could only increase its size or die, and the process of cellular survival was ensured by the continual replacement of intracellular organelles.

Careful, reproducible, and widely reported experiments have overthrown this static-organ theory. Convincing evidence suggests that cardiomyocytes are renewed throughout life, with continual cellular turnover in the mammalian heart, which also has an intrinsic regenerative capacity (Barile et al., 2007). However, the precise rate of cardiomyocyte turnover remains unclear (Ellison et al., 2013). This rate is difficult to study in humans because, in most cases, the necessary methodology involves the use of analogous nucleotide markers (Bergmann et al., 2010). Nuclear bomb testing during the Cold War resulted in integration of ^14^C into cellular DNA, making it possible to determine the age of cardiomyocytes in humans based on analysis of the natural radioactive isotype of carbon to estimate their turnover rate (Bergmann et al., 2010). These studies suggested an annual turnover rate of 0.2–2%; however, a clear negative correlation between the cellular renewal rate and age was observed (Bergmann et al., 2010).

In addition, the source of cardiomyocyte renewal throughout life is unknown (Chong et al., 2011). At least two different origins of these cells must be considered when examining the stimuli that regulate the renewal of cardiomyocytes. As early as, Anversa and Kajstura (1998) proposed that preexisting cardiac cells can divide both under physiological conditions and in response to cardiac overload. Subsequent detailed *in vitro* studies, supported by video microscopy, confirmed the mitotic capacity of cardiomyocytes, particularly mononucleated cardiac myocytes, despite their complex organization (Bersell et al., 2009). At baseline, the mitotic capacity is quite limited, but a considerable proportion of mitotic cardiomyocytes are observed in ischemic hearts and, compared to normal hearts, infarcted hearts have 70 times as many myocytes undergoing mitosis within the border zone (Beltrami et al., 2001).

The second source of mitotic cardiac cells considers the role of cardiac stem cells (CSCs). In 2003, the heart was shown to be regulated by its own pool of stem cells (Beltrami et al., 2003), which established the role of these multipotent cells in regulating the rate of cellular turnover and preserving organ homeostasis.

## Cardiac stem cells

**Figure 1:**
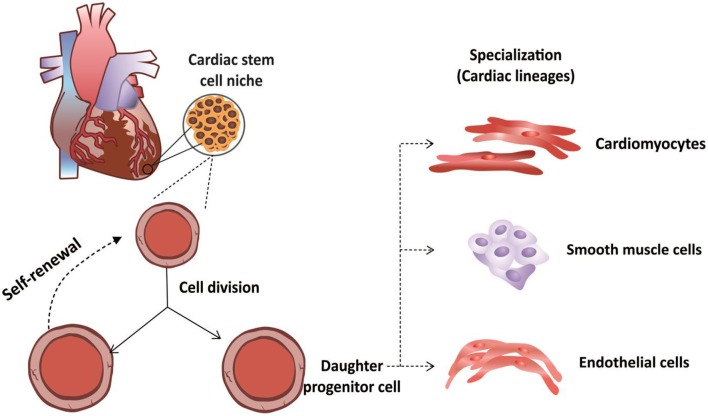
**Functional properties of cardiac stem cells**. Cardiac stem cells are not differentiated cells and can divide without limitation. During cellular division, these cells can divide through symmetrical division to increase their numbers. Alternatively, these stem cells can undergo asymmetrical cellular division to produce both a daughter stem cell and a progenitor cell, the latter of which can differentiates along the three major cardiac lineages: cardiomyocytes, endothelial cells or smooth muscle cells.

CSCs were first isolated by Beltrami et al. (2003) and characterized as a population of cells that were positive for the c-kit surface receptor (Di Felice et al., 2009). In addition to the presence of this receptor, CSCs exhibit clonogenic and self-renewal capacities and multipotentiality, allowing them to differentiate along the three main cardiac lineages: myocytes, endothelial cells and smooth muscle cells (Di Felice et al., 2009) (Figure 1).

In addition to c-kit, other specific phenotypic markers define other “types” of CSCs, although some of these markers may be co-expressed by some cells. CSCs of particular interest include (i) c-kit^+^; (ii) side population cells; (iii) Sca-1^+^; (iv) Isl1^+^; and (v) CSCs derived from cardiospheres (Chan et al., 2009). These CSCs all exhibit properties consistent with “real stem cells,” including the following: (i) a lack of complete differentiation; (ii) the ability to divide without limitation; (iii) symmetrical division to generate two daughter stem cells to expand the stem cell compartment of the heart, i.e., self-renewal, or even asymmetrical to generate one daughter stem cell and a cell bound to a specific cellular lineage (Urbanek et al., 2006; Kajstura et al., 2010b) that subsequently undergoes terminal cellular differentiation (Raff, 2003; Leri et al., 2005).

## c-Kit^+^ cardiac stem cells

c-Kit^+^ CSCs are undifferentiated cells whose *in vitro* and *in vivo* properties are essentially identical and indistinguishable between species (Ferreira-Martins et al., 2012). c-Kit is a transmembrane receptor for a tyrosine kinase factor, and its ligand–stem cell factor (SCF)–is an early hematopoietic growth factor (Chen et al., 2013). c-Kit^+^ cells are the most widely studied CSCs. These cells are one-tenth the size of cardiomyocytes and may express cardiac-specific-lineage transcription factors such as Nkx2.5, GATA4, and Mef2 (Beltrami et al., 2003; Barile et al., 2007). Their transcriptional profile indicates that c-Kit^+^ cells are the most primitive population present in the heart and may play a role in early mesodermal development and stem-cell signaling pathways (Dey et al., 2013). Because the c-Kit receptor is also expressed by various differentiated adult cells, such as mast cells (Fang et al., 2012), in addition to being positive for c-kit, CSCs must also be negative for various cell-specific lineage markers (e.g., c-Kit^+^Lin^−^).

c-Kit^+^Lin^−^ CSCs are found in small clusters in the interstices between well-differentiated myocytes, in which it is possible to observe cells at several stages of early cardiac myogenic differentiation based on their expression of the characteristic transcription factors, allowing one to infer that these clusters represent myogenic precursors and progenitor cells derived from the activation of more primitive stem cells (Beltrami et al., 2003).

Human cardiac homeostasis is closely related to β-adrenergic signaling throughout life, and an important study demonstrated the existence of a direct relationship between the β-adrenergic receptor system and c-Kit^+^ CSCs (Khan et al., 2013). c-Kit^+^ CSCs exhibit cardiac β2-adrenergic receptors at their membranes; and the function assigned to them is to stimulate multipotent CSCs to proliferate via the ERK-Akt pathway (Khan et al., 2013).

When isolated c-Kit^+^ cells are grown in suspension, they form spheres containing hundreds of cells similar to the pseudo-embryoid bodies formed by neural stem cells (neurospheres). By analogy, these spheres were called cardiospheres and represent a population of multipotent cells with distinct characteristics (Torella et al., 2007).

## Cardiosphere-derived stem cells

Initially isolated by Messina et al. (2004), cardiosphere-derived cells (CDCs) can differentiate into myocytes, endothelial cells and smooth muscle cells (Li et al., 2010). CDCs have been characterized as clonogenic cells, and they express markers of stem cells and endothelial progenitor cells (Messina et al., 2004). Stem cells derived from cardiospheres in a three-dimensional configuration exhibit a higher rate of c-Kit expression and increased expression of Nanog and SOX2, two important stem-cell transcription factors (Li et al., 2010).

A recent report investigating stem cell surface markers showed that CDCs are CD105^+^ stromal cells of intrinsic cardiac origin that include a variable fraction of CD90 (Thy-1)-positive cells and a small minority of c-kit^+^ cells, which have been argued to represent cardiac progenitors (Cheng et al., 2014). A clinical trial in which CDCs were transplanted into infarcted areas revealed that the c-kit^+^ cells in cardiospheres are not an important determinant of the therapeutic efficacy of CDCs, whereas CD90^+^ cells appear to undermine the overall benefit of CDC therapy (Cheng et al., 2014). Thus, c-kit expression on CDCs may be irrelevant to the therapeutic efficacy of this stem cell type. Moreover, the dissociation of cardiospheres into single cells appears to decrease their expression of extracellular matrix and adhesion molecules and affect their resistance to oxidative stress, negating the improved cellular engraftment and functional benefit of CDCs *in vivo* (Li et al., 2010).

## Sca-1^+^ cardiac stem cells

Sca-1^+^ cells, which express the surface marker stem cell antigen 1, were initially isolated from mouse hearts by Oh et al. (2003) and Matsuura et al. (2004), who demonstrated that these cells can differentiate into distinct cell lines *in vitro* (Di Felice et al., 2009). This differentiation ability is retained even after long-term propagation *in vitro* (Wang et al., 2014). Sca-1 is a convenient marker for stem-cell studies because it is expressed by stem/progenitor cells and its expression is upregulated in a variety of murine cancer stem cells. Recent studies have identified Sca-1^+^ in various tissues, such as the mammary gland (Welm et al., 2002), prostate (Xin et al., 2005), dermis (Toma et al., 2005), skeletal muscle (Gussoni et al., 1999), heart (Rosenblatt-velin et al., 2005), and liver (Wulf et al., 2003). However, in all cases, it is unclear if the Sca-1^+^ populations are tissue-specific precursor/stem cells or are the hematopoietic, mesenchymal, or endothelial precursor/stem cells associated with these tissues (Holmes and Stanford, 2007). The Sca-1 marker is used to characterize CSCs only in association with other specific markers to exclude differentiated cells and cells of other lineages.

Cardiac Sca-1^+^ cells are capable of cardiac repair and have high levels of telomerase. These cells have the ability to migrate and home to areas of injured myocardium (Oh et al., 2003). *In vitro*, they can be propagated for a long period without any significant changes in marker expression (Wang et al., 2014). Molecular identification studies have indicated that Sca-1^+^ cells are more strongly correlated with cardiomyocytes compared to c-Kit^+^ cells (Dey et al., 2013). Moreover, Sca-1^+^ cells obtained from adult hearts express GATA4 and Csx/Nkx-2.5, suggesting that they are committed to the cardiomyocyte lineage to some degree (Matsuura et al., 2004).

Some Sca-1^+^ cells are committed to the cardiomyogenic lineage before birth; subsequently, Sca1-derived cells play a significant role by contributing continuously to cardiomyogenesis (Uchida et al., 2013). However, it appears that Sca1^+^ cells are more responsive to inductive cues that promote differentiation into cardiomyocytes in healthy compared to diseased myocardium (Uchida et al., 2013), which might explain the limited but important contribution of Sca-1^+^ CSCs to the adult myocardium. Recent findings partly corroborate this theory, demonstrating the relevant role of endothelial cells in generating cardiomyocyte progeny in the adult heart, with direct involvement of cardiac Sca-1^+^ cells in this process (Fioret et al., 2014). Lineage tracing experiments have revealed endothelial progeny in perivascular areas, including Sca-1^+^ cardiac progenitor cells, suggesting that a significant fraction of Sca1^+^ CSCs are descendants of endothelial cells (Fioret et al., 2014). These Sca-1^+^ cells proliferate, leave the coronary niche, and differentiate into cardiomyocytes, which indicates that the coronary vessels serve as an Sca-1 cell niche and further suggest that a negative impact on this niche environment evoked by any type of insult to coronary vessels could affect the proliferation and differentiation of CSCs (Fioret et al., 2014).

## Side-population stem cells

Another phenotypic class of CSCs is side population cells and was initially discovered by Hierlihy et al. (2002) based on their identification of cells sensitive to verapamil in the adult human myocardium. These cells are characterized by their ability to promote the efflux of metabolic markers, such as Hoechst dye, due to high expression of membrane pumps and genes conferring resistance to multiple drugs (Hierlihy et al., 2002; Martin et al., 2004). This efflux ability is due to their expression of the Abcg2 protein, which belongs to the family of transporters linked to the ATP cassette (ABC transporters), early during cardiac development, which endows these cells with their characteristic phenotype (Di Felice et al., 2009). Under pathological conditions, human Abcg2 mRNA levels increase, suggesting the possibility of the activation and proliferation of side population cells in response to cardiac diseases in an attempt to promote the reparative potential (Smith et al., 2008b). Increased numbers of cells expressing Abcg2 have also been detected in the marginal zone of acute infarcted myocardium (Barile et al., 2007).

When co-cultured with cardiomyocytes, side population cells express markers of terminal differentiation and exhibit the electrophysiological characteristics of differentiated cardiac myocytes, including their response to β-adrenergic agonists (Barile et al., 2007). Under physiological conditions, side population cells can be isolated only from the hearts of very young mammals, and the number of these cells decreases rapidly during the first weeks of life (Barile et al., 2007). Approximately 93% of side population cells are also positive for Sca-1 (Oh et al., 2003).

## Isl1^+^ stem cells

The characteristic marker of Isl1^+^ cells is the LIM-homeodomain transcription factor Islet-1 (Laugwitz et al., 2005). Some of the distribution features of these cells suggest that Isl1^+^ cells are organ-specific progenitor cells remaining from the fetal progenitor population (Laugwitz et al., 2005). Murine or human Isl1^+^ cells derived from embryonic stem cells have been shown to give rise to cardiomyocytes, which exhibit appropriate electrophysiological properties, such as action potentials and Ca^2+^ transients (Cagavi et al., 2014), as well as smooth muscle cells and endothelial cells (Lui et al., 2013). Contrary to previous assumptions, Isl1^+^ cells are not exclusively in an embryonic state because there is a subpopulation of endogenous CSCs that persist throughout life and co-express both c-kit and isl1 (Fuentes et al., 2013). Because all progenitor cells expressing Isl1 also express c-kit but not all c-kit^+^ cells express Isl1, Isl1^+^c-kit^+^ progenitor cells may be a subpopulation of c-kit^+^ progenitors (Fuentes et al., 2013).

Comparisons of embryonic and adult Isl1^+^ cells have revealed that their significantly different levels of proliferative and migratory abilities are a consequence of age (Fuentes et al., 2013). Cardiac progenitor cells derived from embryonic stem cells may be a valid source for cell-based cardiac-repair therapies because their use would address the age-related decline in cardiac progenitor cell function; furthermore, the potential risk of teratoma formation and genomic alterations attributed to embryonic cells is minimal for Isl1^+^ cells because they are highly committed cardiovascular cells (Cagavi et al., 2014).

## Cardiac mesenchymal stem cells

Another class of multipotent cells present in the heart are cardiac mesenchymal stem cells (cMSCs), which have been referred to in the literature as cardiac mesenchymal stem-like cells (Ryzhov et al., 2014) and cardiac mesenchymal-like stromal cells (Vecellio et al., 2012). MSCs were initially derived from the plastic-adherent fraction of components of mononuclear cells isolated by density-gradient centrifugation of bone marrow cells and culturing on an adherent surface (Friedenstein et al., 1974). MSCs have since been derived from many organs other than bone marrow (da Silva Meirelles et al., 2006) and were recently, identified in the cardiac stroma (Chong et al., 2011).

The phenotypic characterization of this cell type is complex, and there is not a specific marker or combination of markers to identify MSCs (Javazon et al., 2004). In culture, the phenotype of MSCs is altered; the surface markers of freshly isolated mesenchymal cells differ from those maintained in culture for a long period (Eggenhofer et al., 2014). In addition to the absence of a “phenotypic marker,” the characteristics of MSCs differ depending on the species of origin (Javazon et al., 2004). Therefore, MSCs are usually defined based on a combination of physical, morphological, phenotypic, and functional properties (Javazon et al., 2004; Le Blanc and Ringdén, 2005). The following minimal criteria have been established for the identification of MSCs: (i) adherence to plastic; (ii) adipogenic, chondrogenic, and osteogenic differentiation capacities; (iii) expression of CD73, CD90 and CD105 and the absence of surface markers such as CD45, CD34, CD11b and CD14, CD79α or CD19 and HLA-DR.; and (iv) ability to generate colony-forming unit fibroblasts (CFU-Fs) (Dominici et al., 2006; Gambini et al., 2011).

In contrast to other stem cells present in the heart, MSCs lose their multipotentiality with passage in culture and enter senescence (Javazon et al., 2004), which means that the growth potential of these cells is limited. Bulk cultures of freshly isolated cardiac CFU-Fs grow exponentially for approximately 40 passages before entering senescence (Chong et al., 2011). Despite this limitation, these cells have important regenerative, angiogenic, antiapoptotic, and immunosuppressive properties (Nora et al., 2012).

MSCs can suppress monocyte differentiation into dendritic cells and down-regulate the expression of costimulatory molecules by mature dendritic cells, thereby decreasing their secretion of IL-12 and suppressing T-cell activation and proliferation (Jiang et al., 2005). In addition, MSCs regulate the function of macrophages by polarizing these cells preferentially to the M2 anti-inflammatory phenotype and consequently creating an environment favorable for accommodating therapeutic MSCs (Cho et al., 2014).

In the heart, cardiac MSCs are negative for c-Kit and positive for both pericyte (CD146^+^) and fibroblast markers (vimentin and human fibroblast surface antigen) and exhibit features similar to those of syngeneic MSCs extracted from bone marrow, such as comparable morphology and the expression of mesenchymal antigens (CD105, CD73, CD29, and CD44) (Vecellio et al., 2012). However, comparison of bone marrow and cardiac CFU-Fs revealed distinct lineage signatures, indicating that they arise from different progenitor beds during development (Chong et al., 2011). Thus, a preferable differentiation capacity appears to develop depending on the source of the MSCs (Kern et al., 2006).

Thus, given the criteria of each CSC “type” and the variety of primitive cells existing in the heart, it is unlikely that these CSCs perform the same biological functions (Dey et al., 2013). It remains to be elucidated whether phenotypically different CSCs are different types of undifferentiated cells originating from different precursors (Wang et al., 2014) or only represent different developmental stages of the same generic CSC (Torella et al., 2007; Ellison et al., 2013), with only modest variations in phenotype (Ferreira-Martins et al., 2012).

## Organization and distribution of resident stem cells in the heart

In the heart, the CSCs are organized in niches that are preferentially allocated in the atrium and ventricular apex, areas that are protected because they are exposed to low levels of hemodynamic stress (Leri, 2009). These niches are connected by supporting cells, such as fibroblasts and myocytes, highlighting the importance of connexins and cadherins, which play roles in the formation of gap junctions and adherens junctions at the interfaces of these different cell types (Barile et al., 2007; Ferreira-Martins et al., 2012). These cardiac niches create the micro-environment necessary for the long-term residence, survival, and growth of CSCs (Urbanek et al., 2006), providing nutrition and enabling them to maintain tissue homeostasis (Barile et al., 2007). The homeostasis of these niches is mediated by symmetric and asymmetric mitosis, with a predominance of asymmetric mitosis, which ensures the protection of the pool of stem cells and the production of lineage-committed cells (Urbanek et al., 2006; Barile et al., 2007).

On the other hand, cardiac MSCs are located near pericytes and are widely distributed in different organs in association with vessel walls (da Silva Meirelles et al., 2006). In the heart, MSCs occupy a perivascular, adventitial niche and may represent a population of progenitor cells that are able to maintain the integrity of the matrix, stroma, and vessels of the heart and contribute to the parenchyma, particularly in cases of injury and disease (Chong et al., 2011).

## Extracardiac stem cells in the heart

Stem cells other than resident CSCs are found in the heart. Evidence for the presence of mobile stem cells in the heart was provided by human heart transplantation experiments in which a significant number of Y-chromosome positive myocytes and coronary vessels were observed in the transplanted heart when the donor was female and the recipient was male (Quaini et al., 2002). In this case, the male cells in the female heart suggested the existence of mobile stem cells that were capable of differentiating into cardiomyocytes, endothelial cells or vascular smooth muscle cells (Beltrami et al., 2003). When the origin and population dynamics of cardiac progenitor cells were investigated using a heterotopic mouse heart transplantation model, cells that migrated to the heart after transplantation gradually ceased expressing the hematopoietic markers CD45 and CD34 and began to express cardio-specific transcription factors such as GATA-4 and Nkx2.5, hence acquiring a cardiac-cell phenotype (Li et al., 2007). Conditions other than organ transplantation that have the potential to attract circulating stem cells and the potential sources of these cells are being investigated.

Hematopoietic stem cells (HSCs) reside predominantly in the bone marrow but can be found in the peripheral blood in low numbers (Wright et al., 2001) and in the spleen, which is considered an active hematopoietic organ because it serves as a reservoir for hematopoietic stem cells for relatively immediate use (Morita et al., 2011). Little is known about the physiological role of these HSCs in normal adult tissue homeostasis (Zhang et al., 2006), although it has been described that HSCs have the ability to acquire a cardiomyocyte phenotype (Orlic et al., 2001; Rota et al., 2007).

The chemokine SDF-1α (stromal-derived factor-1) has a well-described role in the subsequent migration and homing of stem cells. SDF-1α belongs to the subfamily of chemokines that can facilitate the transmigration of hematopoietic cells through the endothelial cell barrier (Chen et al., 2013). In an infarction model, the attraction exercised by SDF-1 is not homogeneous for the different types of stem cells because more c-kit^+^ than Sca-1^+^ cells were recruited, for example (Zhang et al., 2007). This chemokine also participates in the recruitment of resident CSCs in specific situations by binding to the CXCR4 molecules of these cells (Zakharova et al., 2010; Wang et al., 2012; Yan et al., 2012; Chen et al., 2013; Ellison et al., 2013). The up-regulation of CXCR4 expression increases the vascularization of the injured myocardium, and the SDF-1/CXCR4 axis appears to be particularly important in the chemotaxis, homing, engraftment and attachment of progenitor cells to the injured myocardium (Wang et al., 2006b).

Hypoxic preconditioning increases the expression of CXCR4 in c-Kit^+^ cells, which indicates that this response is related to the promotion of cellular survival achieved by activating the SDF1-α/CXCR4 axis (Yan et al., 2012). Furthermore, the rapid increase in chemokines/cytokines such as SDF-1α contributes to the upregulation of MMP-9 (matrix-metalloproteinase 9) expression, which is involved in the recruitment of hematopoietic stem cells (Heissig et al., 2002). Matrix metalloproteinases are a family of endopeptidases that act as physiological regulators of the extracellular matrix (Shah et al., 2011). For example, upon cardiac injury, the activation of c-kit^+^ cells via an MMP-9 dependent pathway is required to mobilize progenitor cells from bone marrow to home to the heart (Fazel et al., 2008). In an acute myocardial infarction model, an increase in MMP-9 levels was observed, which may represent an endogenous mechanism for the recruitment of undifferentiated stem cells to the area of myocardial injury (Shah et al., 2011).

It is not clear if the cells attracted to the injured heart due to chemokine stimulus or stimulation by other factors released by the remaining heart cells or, in the absence of injury, due to the growth factors released by the heart are able to acquire a differentiated cardiac-cell phenotype. Using parabiotic pairs of animals, Wagers et al. (2002) observed that despite high levels of cross hematopoietic grafting, under physiological and stable conditions, multiple tissues showed no evidence of grafting of the circulating hematopoietic stem cells on non-hematopoietic tissue (Wagers et al., 2002). These findings suggested that hematopoietic cells do not develop into non-hematopoietic cells; however, the authors could not rule out atypical properties arising in response to severe injury or selective pressure (Wagers et al., 2002). Their theory was that at steady state, tissue regeneration appears to derive predominantly from progenitor cells resident in the tissue rather than from circulating cells (Wagers et al., 2002). Subsequently, to investigate cellular migration in response to pathological stimulus, Balsam et al. (2004) performed a left anterior descending coronary-artery ligation immediately after parabiosis surgery in mice. After 8 weeks, large numbers of GFP^+^ cells were observed surrounding the scar tissue; however, these cells co-expressed CD45, some co-expressed cell marker B (B220), a few co-expressed a T-cell marker (CD3), and none expressed cardiac or smooth muscle markers (Balsam et al., 2004). These results suggest that even after cardiac injury, circulating cells do not regenerate myocardium. However, the descending artery was ligated immediately after the animals were joined, and circulatory chimerism was not yet established, which may have led to an underestimation of the cells that migrated to the lesion foci (Aicher et al., 2007).

**Figure 2:**
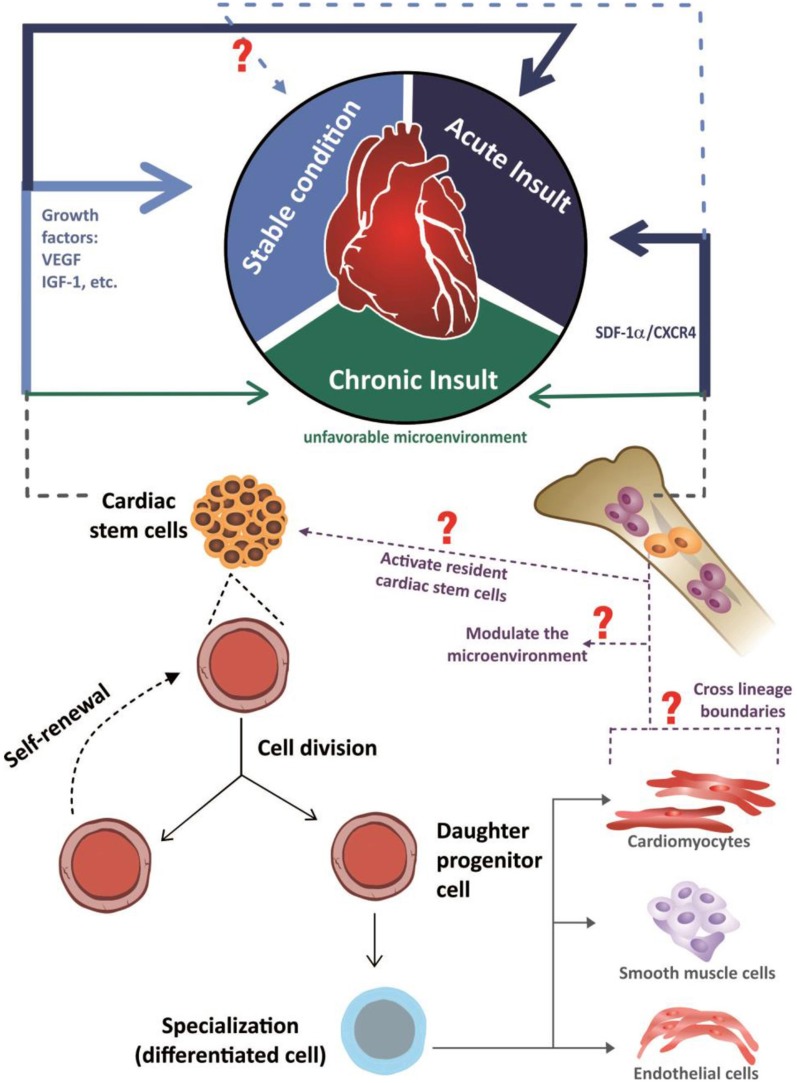
**Schematic representation of the mechanisms through which stem cells are involved in both cardiac homeostasis and in the heart repair**. In response to acute insult, the damaged myocardium elicits the activation of cardiac and also extracardiac stem cells (represented by the continuous dark blue arrows). The cardiac stem cells are activated in response to growth factors, like VEGF (vascular endothelial growth factor) and IGF-1(insulin-like growth factor 1), while extracardiac stem cells (schematically represented by hematopoietic bone marrow cells) are chemo- attracted into the injured sites through the axis SDF-1α/CXCR4 (stromal-derived factor-1/receptor CXCR4). With the insult chronicity, the participation of cardiac and extracardiac stem cells in the damaged heart is reduced, possibly due to an unfavorable microenvironment to the action of these cells (represented by the continuous dark green arrows). Considering the uninjured heart, in a stable condition, the available information indicates that cardiac homeostasis is ensured by an active role of cardiac stem cells (represented by the continuous light blue arrow), with an unclear role of extracardiac stem cells (represented by light blue dashed arrow). Seeing both acute, chronic and stable conditions, in response to environmental stimuli, cardiac stem cells differentiate into cardiomyocytes, endothelial cells or smooth muscle cells and also, possibly, release soluble autocrine/paracrine factors that play roles in both stem cells self-renewal and myocardial protection/neovascularization respectively, while extra cardiac stem cells are involved in the beneficial modulation of the microenvironment, since the capacity of them to acquire a differentiated phenotype, crossing lineage boundaries, is questionable.

In another model used to investigate stem-cell migration, mice were subjected to irradiation, and bone marrow cells were replaced by eGFP^+^ bone marrow cells. After cardiac injury, several types of histological analysis revealed that no cardiac cells with fluorescent-protein expression were present in the heart, indicating that stem cells derived from the bone marrow do not directly contribute to cardiomyocyte formation in injured or uninjured hearts (Ellison et al., 2013). Thus, the currently available evidence indicates that the resident or organ-specific stem cells contribute to the maintenance of tissue homeostasis, whereas the non-resident circulating stem cells that are attracted to organs minimally contribute to this physiological process, highlighting the restorative activities of these cells when attracted to an organ by certain stimuli (Figure 2), which was first demonstrated in 2002 via chimerism in a post-transplantation organ (Laflamme, 2002; Quaini et al., 2002; Angelini et al., 2007).

## Cardiac stem cells under homeostatic or unstable conditions

In addition to contributing to the maintenance of tissue homeostasis (Ferreira-Martins et al., 2012; Waring et al., 2014), CSCs participate in reparative processes under pathological conditions (Bailey et al., 2009; Ellison et al., 2013). However, extensive damage cannot be completely reversed, i.e., the regenerative potential of stem cells in damaged tissues is limited (Bailey et al., 2009; Chan et al., 2009; Hatzistergos et al., 2010). Therefore, massive degenerative events such as myocardial infarction cannot be counteracted by resident CSCs (Smith et al., 2008b).

Under physiological conditions, the pool of resident c-kit^+^ CSCs present during early cardiac development were suggested to be responsible for expanding the myocyte mass during embryonic, fetal, and immediate postnatal development (Ferreira-Martins et al., 2012). However, the results of a recent study overturned this assumption, by demonstrating, using a very elegant model and analyzing different stages of cardiac development as well as the postnatal period, that the c-kit^+^ CSCs contributed minimally to cardiomyocyte formation (van Berlo et al., 2014). By contrast, these cells play a significant role in the origin of endothelial cells (van Berlo et al., 2014). Furthermore, the physiological role of c-Kit^+^ CSCs includes paracrine activity. *In vitro* experiments revealed an increased rate of cardiomyocyte survival when c-Kit^+^ CSCs were co-cultured with adult rat cardiomyocytes, and the secretion of growth factors, such as IGF-1 (insulin-like growth factor 1) and VEGF (vascular endothelial growth factor), appeared to be responsible for the beneficial effect (Miyamoto et al., 2010). In addition, with respect to cardiac repair after heart injury, c-Kit^+^ CSCs can acquire a cardiomyocyte phenotype (Ellison et al., 2013), highlighting the different activities of these multipotent cells under physiological and pathological conditions.

Certain conditions can lead to functional impairment of CSCs. In the pathological heart, the homeostatic control of stem cell growth is defective, resulting in cellular senescence (Chimenti et al., 2003; Urbanek et al., 2005) and apoptosis (Urbanek et al., 2005). Thus, although the number of CSCs increases after an ischemic cardiac event, the level of regeneration is not in the expected range, which is at least partially attributable to the acquisition of a senescent phenotype by many CSCs in infarcted hearts. These cells thus lose their regenerative capacity and become non-cycling, non-differentiating cells (Urbanek et al., 2005).

Functional problems also occur in CSCs in chronic systemic disease, and these changes have implications for the heart. Diabetes leads to decompensated myopathy, an etiology that is poorly understood. Because the heart constantly renews itself, an imbalance between cellular death and regeneration may occur in diabetes, and this imbalance may be mediated by defects in the growth and survival of c-Kit^+^ CSCs, which are massively reduced in number, as well as reductions in telomere length (Rota et al., 2006). The impairment in cardiac progenitor cell function as a consequence of the enhanced oxidative stress that occurs with this disease affects cellular turnover, resulting in an excessive number of old, dying, and poorly contracting myocytes and, ultimately, ventricular failure, which in turns may result in the pathological manifestations of diabetic myopathy (Rota et al., 2006). When diabetes is treated with antioxidants, the numbers and functional abilities of both progenitor cardiac cells and mature cardiac cells are preserved, and a marked recovery of ventricular function is observed (Delucchi et al., 2012), supporting the theory that oxidative stress in diabetes leads to the loss of CSCs.

The same damaging effects of oxidative stress appear to be involved in the cardiac injury observed in subjects treated with anti-tumor therapies. Anthracyclines are effective drugs for the treatment of some neoplastic diseases (De Angelis et al., 2010), but they have profound effects on the structure and function of the heart, which over time causes cardiomyopathy that evolves to congestive heart failure (Takemura and Fujiwara, 2007). The cardiotoxicity of the anthracyclines is dose dependent, limiting their aggressive use (De Angelis et al., 2010). *In vitro* studies have demonstrated that anthracyclines promote oxidative stress and the activation of p53, a protein that regulates the cell cycle, thereby affecting the growth and survival of c-Kit^+^ CSCs. This effect supports the theory that defects in progenitor cell function may condition the development of cardiac myopathy *in vivo* (De Angelis et al., 2010; Prezioso et al., 2010). Thus, deleterious cardiac effects with different causes may be due to targeting of CSCs, emphasizing that injury to these cells that contribute to the maintenance of cardiac homeostasis will have inevitable cardiac consequences.

Chronological age is a major predictor of the presence of biomarkers of human CSC senescence such as telomere shortening, attenuated telomerase activity, telomere dysfunction, and the expression of p21^Cip1^ and p16^INK4a^, both of which are senescence-associated proteins (Cesselli et al., 2011), raising questions about the functional capacity of cells taken from aging hearts and hearts in chronic failure. Comparative analyses of CSCs isolated from transplanted hearts (both donor and explanted hearts) have revealed that the clonogenic capacity of CSCs in the heart in heart failure is three times lower than that of cells from healthy hearts. By contrast, the differentiation potentials of CSCs from healthy and infarcted hearts were comparable and yielded similar proportions of myocytes, smooth muscle cells, and endothelial cells, indicating the possibility of autologous transplantation in individuals with heart failure (Cesselli et al., 2011). Thus, even in decompensated hearts, there is a compartment of functionally competent CSCs that, despite lower growth kinetics, retain the ability to expand and differentiate. These characteristics are promising for the clinical application of these cells (Cesselli et al., 2011).

MSCs are affected by aging and exhibit diminished expression of Nanog, a transcription factor that retains MSCs in a primitive state, as well as increased adipocytic potential (Cieslik et al., 2011). Furthermore, fibroblasts derived from aged MSCs exhibit reduced expression of transforming growth factor-β (TGF-β) receptors, attenuated contractility and migratory ability (Cieslik et al., 2011), and decreased expression of α-SMA, an indicator of poor maturation into myofibroblasts (Cieslik et al., 2013). In addition, the fibroblasts derived from elderly MSCs exhibit increased expression of type I collagen due to the elevation of circulating plasma insulin levels with aging (Cieslik et al., 2013). Future *in vivo* studies using pathological models treated with cardiac MSCs isolated from animals of different ages could be able to reveal possible differences in the functional abilities of aged cells.

## Cellular therapy with stem cells as a treatment option

A massive number of cardiomyocytes die after a heart attack, and the basic concept for cellular therapy following myocardial infarction must consider this large-scale cellular loss resulting in heart failure (Lovell and Mathur, 2011). Therefore, treatments based on stem cell transplantation aim to avoid the loss or failure of cardiovascular function (Sánchez et al., 2010) and to rescue the numbers of cardiomyocytes to ensure good cardiac performance (Noseda and Schneider, 2010). It is important to repair not only the cardiomyocytes but also the endothelial and smooth muscle cells, which are crucial for developing a new network of arteries to bring nutrients and oxygen to the cardiomyocytes and restore organ function after heart damage. In the acute phase of a myocardial infarction (MI), the goals of cellular therapy are to prevent cardiomyocyte death, promote local neoangiogenesis, improve myocardial perfusion, and reduce local inflammatory responses (Takashima et al., 2013). Another major objective of an ideal clinical intervention is to avoid scar formation or replace scar tissue with functioning cardiac muscle tissue (Curtis and Russell, 2010).

Orlic et al. (2001) demonstrated that c-Kit^+^Lin^−^ bone-marrow cells could regenerate the infarcted myocardium by demonstrating that the newly formed myocardium occupied 68% of the infarcted portion of the ventricle 9 days after transplanting the bone marrow cells. However, these results were not reproduced by other researchers, who consistently demonstrated that hematopoietic stem cells did not readily acquire a cardiac phenotype (Balsam et al., 2004; Murry et al., 2004). This discrepancy was attributed to the assays used to detect cardiomyogenic differentiation, which was exclusively based on immunofluorescence staining and is thus difficult to use in tissues with high levels of non-specific autofluorescence, as is typically encountered in the infarcted heart (Murry et al., 2004).

However, despite the absence or extremely low rate of acquisition of a cardiac phenotype, experimental studies and, to a lesser extent, clinical studies (Schächinger et al., 2006; Tendera et al., 2009; Hare et al., 2012) have investigated the use of stem cells derived from bone marrow in cases of cardiac injury. Optimistic results have been reported, such as an acceleration of left ventricular ejection fraction (LVEF) recovery after acute myocardial infarction, although this was observed without a long-term benefit on left ventricular systolic function measured at 18 months after bone marrow cells transfer (Meyer et al., 2006). However, a trend in favor of this cell therapy is defended, particularly considering patients with severely impaired LVEF at baseline (Tendera et al., 2009). The favorable results arising from the transplantation of bone marrow cells have been attributed to humoral (Cho et al., 2007) and paracrine effects rather than the direct regeneration of various cardiac cells through transdifferentiation (Loffredo et al., 2011).

Importantly, bone marrow-derived stem cells are easy to obtain in sufficient numbers for transplantation, which hastens the transplantation process because it is not necessary to seed these cells in culture to expand their number. However, because the ability of this cell type to differentiate to replace dysfunctional cardiac tissue is limited, other stem cells with this capability have been sought. Thus, criteria for the ideal cell type for the promotion of consistent improvements in the damaged myocardium with a real potential to replace lost cells have been established. These cells should be capable of differentiating into functional cardiomyocytes and of forming new vessels (Sánchez et al., 2010), indicating cardiac commitment, and they should be able to integrate with the target tissue by developing gap junctions with host cells without inducing immune reactions. Lastly, from a practical standpoint, these cells should preferably exhibit some degree of resistance to ischemia to avoid massive apoptosis during cellular transfer (Gaetani et al., 2009).

Thus, among the types of stem cells studied to date, cardiac progenitor/stem cells appear to represent an attractive option for use in clinical trials because they are intrinsically more likely to possess all of the characteristics required to repair the damaged heart (Lovell and Mathur, 2011) and improve cardiac function after myocardial injury (Torella et al., 2007).

## Cellular therapy with cardiac stem cells–experimental models

Initial studies of CSCs considered the potential for tropism to the site of cardiac injury but demonstrated that all regenerating cells were located in the necrotic area and not the spared myocardium, even though the cells were injected at the border between the ischemic and healthy myocardium (Beltrami et al., 2003). The injured myocardium provides a *milieu* that supports the homing, nesting, survival, and differentiation of CSCs (Ellison et al., 2013). Furthermore, cardiac tropism is CXCR4-SDF-1 axis-dependent, and the enhanced expression of SDF-1 in the surviving myocardium serves as a positive chemotactic agent (Ellison et al., 2013), activating the ligands of the CSCs themselves through auto/paracrine feedback (Torella et al., 2007).

Therefore, the potential of CSCs as a convenient source for autologous stem-cell therapy has been considered for clinical trials of myocardial regeneration (Gaetani et al., 2009). One of the main advantages of autologous transplantation is the elimination of the potential for a potent immunological response against the allograft in the host. However, CSCs are significantly more difficult to harvest and isolate than bone marrow-derived mononuclear cells (Lovell and Mathur, 2011). Furthermore, the number of cells that can be obtained from an individual is quite small; however, these cells can be expanded in culture (Gaetani et al., 2009). Thus, it is feasible that cells isolated from very small fragments of human myocardium and expanded many-fold *in vitro* could reach numbers appropriate for *in vivo* transplantation in patients while retaining their differentiation potential (Messina et al., 2004).

Among the different stem cells types already described, c-kit^+^ CSCs as well as cells derived from cardiospheres have been more frequently utilized in experimental and clinical studies because they are cell populations with well-defined characteristics and extraction and expansion methods. Moreover, compared to the other adult stem cells in the heart, they appear to be in the most primitive stage of differentiation.

Experimental models of myocardial infarction have demonstrated satisfactory responses after the administration of CSCs, such as an improvement of myocardial remodeling following injury via a combination of a reduction in the cell death rate, induction of endogenous repair mechanisms, and the contribution of new cells to the injured myocardium (Bailey et al., 2009). The results suggested that c-Kit^+^ CSCs promote myocardial regeneration, which contributes to the recovery of the structure and function of the damaged heart (De Angelis et al., 2010).

Studies employing a model of diffuse myocardial damage causing acute heart failure revealed that the extent of myocardial injury is even more pronounced when endogenous c-Kit^+^ CSCs are ablated from the adult myocardium (Ellison et al., 2013). Following the administration of c-Kit^+^ CSCs, cardiac function was restored through the regeneration of the lost cardiomyocytes, smooth muscle cells, endothelial cells, and fibroblasts and even through the maintenance of the stemness of the stem cells (Ellison et al., 2013).

When cells derived from cardiospheres were administrated to transgenic animals with dilated cardiomyopathy, anti-apoptotic, anti-fibrotic and cardioproliferative effects were observed, as well as a decrease in mortality (Aminzadeh et al., 2015). However, the beneficial effects of administering these cells to treat pathological processes were most pronounced when the cells were supplied in the form of cardiospheres, thereby ensuring that a niche similar to that self-developed through these stem-cell structures was maintained (Li et al., 2010).

In an *in vivo* study in which Sca-1^+^ CSCs were transplanted in a mouse cardiac-infarction model, significant angiogenesis was observed in the peri-infarction regions, although with minimal differentiation of the Sca-1^+^ cells into endothelial cells, contrary to the substantial *in vitro* endothelial-cell differentiation capacity of these cells (Wang et al., 2006a). These cells transdifferentiated into cardiomyocytes more frequently, albeit the absolute number of newly differentiated cardiomyocytes was low and most likely insufficient to make substantial direct contributions to left ventricular structure, function, or bioenergetic characteristics (Wang et al., 2006a). However, despite the low rate of transdifferentiation of these cells, transplantation of Sca-1^+^ CSCs attenuated the development of adverse structural, functional, and energetic abnormalities associated with left ventricular remodeling post-myocardial infarction, likely due to paracrine effects (Wang et al., 2006a).

When the effects of bone marrow-derived mesenchymal stem cells were compared to those of mesenchymal stem cells derived from the human heart following direct injection into the myocardium of chronically infarcted rats, the cMSCs survived longer in the tissue, better promoted angiogenesis via paracrine mechanisms, and more efficiently differentiated into cardiomyocytes than the MSCs derived from the bone marrow (Rossini et al., 2011). In addition, combining these cells with other types of stem cells appears to be important. In an animal model, combining stromal cells derived from heart tissue with stem cells derived from cardiospheres resulted in increased survival of this cell group upon transplantation in the infarcted areas, and the cardiac contractile function and extent of replacement of the injured myocardium were optimized because this combination of cells promoted vascularization and cardiomyogenesis (Zakharova et al., 2010).

Furthermore, because the paracrine action exerted by MSCs can be attributed, at least in part, to the extracellular vesicles released by MSCs (previously referred to as microvesicles or exosomes) (Bian et al., 2014), which have the capacity to change the phenotype of injured cells through the horizontal transfer of mRNA, microRNA and proteins, it appears that these cells have the potential to be exploited as an alternative to stem cell-based therapy in novel therapeutic approaches to repair damaged tissues (Biancone et al., 2012). *In vivo*, the extracellular microvesicles derived from bone marrow-derived MSCs elicited neoangiogenesis in ischemic hearts and improved functional recovery in an rat cardiac-infarct model (Bian et al., 2014). Extracts of extracellular vesicles directly isolated from supernatants of cardiac MSCs might be used to treat cardiac injuries with even more promising results, although this topic awaits investigation.

Based on the encouraging results of experimental studies, a rapid transition from promising preclinical experiments to early clinical trials occurred (Lovell and Mathur, 2011), although numerous unresolved questions remain with respect to the underlying molecular mechanisms involved in cell-based therapies (Hoshino et al., 2007). Thus, cellular therapy has advanced rapidly as a treatment option, with some concern within the scientific community that this may have been occurring too quickly and uncritically (Smith et al., 2008a). The nature of this rapid transition meant that early studies considered the safety and feasibility of cellular therapy and were of sufficient power to assess its efficacy; however, these studies were sufficient to provoke early optimism in the medical community, even it was based on the results of small studies (Lovell and Mathur, 2011).

## Cellular therapy with cardiac stem cells–clinical studies

The large body of preclinical evidence motivated human clinical trials of CSCs (Bolli et al., 2011; Chugh et al., 2012). With the primary goal of investigating the safety and feasibility of using autologous CSCs for the treatment of heart failure resulting from ischemic heart disease, the SCIPIO (Stem Cell Infusion in Patients with Ischemic cardiOmyopathy) study, the first study of CSC treatment in humans, received approval to conduct a phase 1 clinical trial using CSCs (Chugh et al., 2012). Methodologically, the investigators used autologous intracoronary infusion of c-Kit^+^/Lin^−^ CSCs derived from auricles that were surgically harvested during coronary artery bypass graft (CABG) in patients with ischemic cardiomyopathy. The LVEF was improved by 8% at 4 months after stem-cell administration, in contrast to no change in the control patients. However, the phase I SCIPIO study was randomized and performed at a single center, and autologous CSCs were administered to patients with severe heart failure secondary to ischemic heart disease (Bolli et al., 2011). In addition, the increase in ejection fraction (EF) was even higher 1 year after the administration of CSCs, suggesting that the CSCs continued to improve left ventricular (LV) function over time, whereas the control group did not exhibit improvement in the EF over time, ruling out any spontaneous temporal benefit (Bolli et al., 2011). Furthermore, this improvement in LV function was coupled with a concomitant decrease in infarct size, which was consistently observed using three different methods of cardiac magnetic resonance and was accompanied by an increase in the LV viable mass, implying the robust regeneration of myocardial tissue (Chugh et al., 2012). The NYHA functional classification also improved in the group that received autologous CSC transplantation, as did their quality of life (Bolli et al., 2011). Thus, the purpose of the SCIPIO study, to investigate the safety of cellular administration, was achieved, and no adverse effects attributable to CSC administration were noted (Bolli et al., 2011), permitting the conclusion that the functional benefits of CSCs were inversely related to the baseline functional status of the myocardial region. That is, the lower the baseline function, the greater the improvement afforded by CSC infusion (Chugh et al., 2012). In addition to the safety of the procedure, this study also demonstrated the feasibility of isolating and expanding CSCs obtained from cardiac tissue during CABG surgery (Chugh et al., 2012).

The CADUCEUS (CArdiosphere-Derived aUtologous stem CElls to reverse ventricUlar dySfunction) clinical trial was another prospective randomized trial of intracoronary infusions of autologous CDCs in patients with recent myocardial infarction and residual systolic dysfunction. The phase 1 trial revealed the feasibility and safety of this therapy, with no major adverse cardiovascular events or cardiac tumor development (Makkar et al., 2012). More importantly, CDCs treatment reduced the extent of the scar mass and increased the level of regional contractility observed at the 6-month follow-up but did not improve the end-diastolic volume, end-systolic volume or the LVEF (Makkar et al., 2012). The final results at the 1-year endpoint did not raise significant safety concerns and proved that, despite improvements in the scar size and regional functionality based on magnetic resonance imaging (MRI)-based analysis of myocardial regeneration and clinical correlates of the regenerative efficacy, no improvements in global function, such as improvements in the NYHA functional class, the peak VO_2_, the distance walked in 6 min or the quality of life after therapy using CDCs, were detected (Malliaras et al., 2014). Covariate analysis revealed that a higher baseline scar size was strongly associated with a greater reduction in scar size 1 year after cellular infusion (Malliaras et al., 2014). Importantly, a patient who underwent late treatment and received the infused cells at 14 months post-MI responded similarly to patients who were infused earlier (at 1.5–3 months post-MI), indicating that CDCs administration might confer benefits to chronic ischemic cardiomyopathic patients similar to those observed in convalescent MI patients (Malliaras et al., 2014).

Another randomized, double-blind, placebo-controlled clinical trial (phase I/II) that is still in progress is using, in contrast to the CADUCEUS trial, allogeneic CDCs instead of autologous cells to achieve myocardial regeneration. The purpose of the ALLSTAR study (ALLogeneic heart STem cells to achieve myocArdial Regeneration) is to investigate the safety and efficacy of the treatment in decreasing infarct size, with the primary efficacy endpoint being the relative percentage improvement in infarct size as assessed using magnetic resonance imaging at 6 and 12 months post-infusion (Allogeneic Heart Stem Cells to Achieve Myocardial Regeneration (ALLSTAR) (NCT01458405)^2^. More recently, a phase I/II randomized, double-blind, placebo controlled clinical trial was proposed by a European study group initiative (CARE-MI) to investigate the therapeutic role of allogeneic CSCs in patients with congestive heart failure due to myocardial infarction (Crisostomo et al., 2015).

Despite the favorable results observed in some individuals treated with stem cells, certain clinical expectations were not achieved because the levels of improvement in cardiac tissue structure or functionality were less than those previously observed in experimental and pre-clinical trials. Although several challenges remain, such as optimization of the therapeutic protocols and technological improvement of stem cell delivery devices, it is believed that the current phase III studies will provide new insights and accelerate the further development and acceptance of cardiovascular regenerative medicine, particularly in relation to reduced mortality following stem cell therapy and the optimal sub selection of stem cells for clinical applications (Takashima et al., 2013; Cheng et al., 2014).

**Figure 3:**
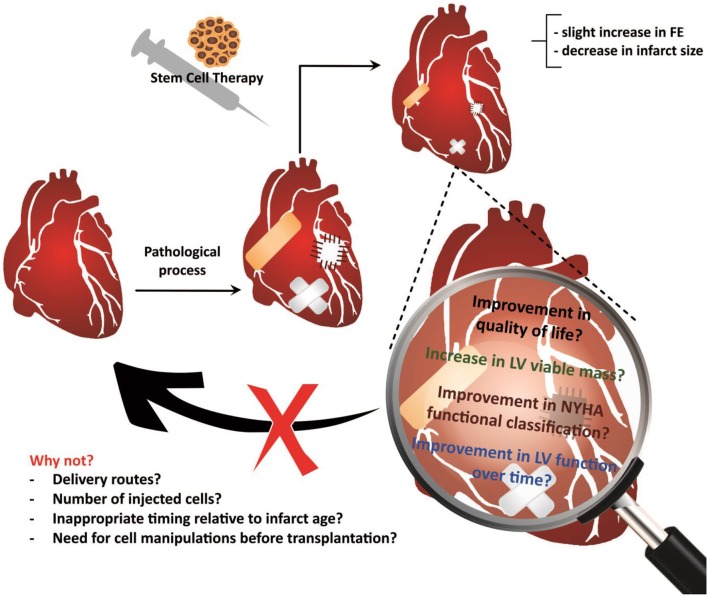
**Treatment using cardiac stem cells and associated outcomes**. Results support the feasibility and safety of the cardiac stem cell delivery procedures, although the reports of outcomes have been limited and primarily concerned to functional improvements or reductions in the infarct size. Future studies will allow investigate other equally important outcomes, such as improvements in the quality of life, maintaining the improvement in ventricular function over time, and decreasing the mortality rate, among others. However, there is still much to be explored in basic studies, which may reveal the reasons why therapy using cardiac stem cells is not able to heal the injured heart.

Although clinical studies using stem cells obtained from different sources for cardiac-repair purposes have generally demonstrated that CSC therapy is safe, it remains unclear why these trials have failed to translate preclinical expectations in humans (Lovell and Mathur, 2011). Various explanations for this discrepancy have been suggested, such as the injection of an insufficient number of cells or the wrong cell type, the use of an inappropriate delivery method, inappropriate timing relative to the age of the infarct, and differences between animal models and humans, including the absence of comorbidities in the animal model (Lovell and Mathur, 2011) (Figure 3).

The results of both clinical and bench studies on this topic demonstrate that there is much that remains to be investigated in this field, particularly regarding the biodistribution of injected cells, the optimal method and route for stem cell delivery, the extent of cellular differentiation of these cells within the target area (Hoshino et al., 2007) the precise paracrine signals that drive the cell fate decision of multipotent progenitors, and the development of novel approaches to deliver these signals *in vivo* (Lui et al., 2013).

## Cellular therapy with cardiac stem cells–future directions

It may be relevant to investigate the effects of administering combinations of stem cells derived from different sources as alternative therapies and to determine the ideal number of these cells to be used, the best timing of injection after damage has occurred, the optimal frequency of injections, and the mechanisms of action of the administered cells, as well as the extents of their homing/grafting and survival and their preferential homing routes (Sánchez et al., 2010). However, many clinical trials have demonstrated small improvements in FE that are sufficient for substantial clinical improvement following stem cell administration, indicating the relevance of this strategy (Lovell and Mathur, 2011). Based on these results, various alternatives are being formulated (Smith et al., 2008a).

*In vitro* studies have indicated the possibility of manipulating stem cells prior to transplantation to optimize their application. For example, when c-kit^+^ CSCs exhibit an increased level of expression of the transcription factor GATA4, their cardiosphere-forming ability is reduced, indicating that their clonogenicity and multipotentiality are also decreased, whereas, their ability to differentiate into cardiomyocytes is increased (Miyamoto et al., 2010). *In vivo* assays are required to confirm these conclusions.

Thus, uncertainties remain about the next steps to advance the promising strategy of cellular therapy (Figure 3). Some have suggested a return to the bench, whereas others support the continuation of clinical practice because there are many examples of new treatments that have been implemented with only a provisional understanding of the underlying mechanisms, followed by elucidation of the mechanisms with clinical experience (Lovell and Mathur, 2011). Supporters of the continuity of clinical studies base their opinions on the fact that, to date, the follow-up period of subjects treated using CSCs is still short, and the vast majority of outcomes investigated were cardiac functional parameters or infarct size. Future studies may provide information about the impact of cellular therapy on the long-term quality of life of treated patients, the possible functional improvements that are maintained over time and the reduction of the rate of or lack of progression of heart failure or of the increase in the late mortality rate following cardiac injury.

The electrophysiological properties of the *in vivo* CSC-derived cardiomyocytes also remain to be investigated (Smith et al., 2008a) because they were demonstrated to be as important in tissue repopulation as the coupling of these newly formed cells to the residual myocyte populations in the heart. The functional integration of these cells was satisfactorily demonstrated in a preclinical study that used an *ex vivo* preparation and two-photon microscopic analysis (Bearzi et al., 2007), but the functional integration of these cells remains to be demonstrated in clinical studies. Likewise, understanding the physiology of the resident CSCs may lead to a better understanding of how to expand and induce these cells to differentiate into functional cardiomyocytes (Di Felice et al., 2009) as well as vascular and endothelial cells within the heart.

To summarize, the perspectives for future studies appear challenging yet encouraging; in particular, the interplay between basic and clinical research will be critical for the development of the field and for achievement of the final goal, which is a safe and efficient therapy utilizing cardiac stem cells to regenerate lost myocardium.

## Conclusions

The currently available data encourage further investigation into the potential application of cardiac multipotent cells to treat the damaged heart. Although CSCs hold therapeutic promise for clinical applicability, much remains to be explored.

## Author contributions

CL drafted the study plan, designed the research plans and wrote the review. TA and CL contributed equally to the scientific research materials and the critical discussion of relevant issues. VS revised the manuscript critically for important intellectual content and coordinated the performance of this study.

### Conflict of interest statement

The authors declare that the research was conducted in the absence of any commercial or financial relationships that could be construed as a potential conflict of interest.

## Footnotes

^1^Available at: http://www.who.int/mediacentre/factsheets/fs355/en/ Acessed January 10, 2015.

^2^Available at: https://clinicaltrials.gov/ct2/show/study/NCT01458405 Accessed January 10, 2015.
